# A health technology assessment of COVID-19 vaccination for Nigerian decision-makers: Identifying stakeholders and pathways to support evidence uptake

**DOI:** 10.1186/s12961-024-01158-y

**Published:** 2024-06-26

**Authors:** Benjamin S. C. Uzochukwu, Chinyere Okeke, Faisal Shuaib, Sergio Torres-Rueda, Anna Vassall, Mark Jit, Justice Nonvignon, Adaora C. Uzochukwu, Francis Ruiz

**Affiliations:** 1 Department of Community Medicine, University of Nigeria Nsukka, Enugu Campus, Nsukka, Nigeria; 2 Health Policy Research Group, College of Medicine, University of Nigeria Nsukka, Enugu Campus, Nsukka, Nigeria; 3 National Primary Health Care Development Agency Abuja, Abuja, Nigeria; 4 Department of Global Health & Development, Faculty of Public Health and Policy, London School of Hygiene and Tropical Medicine, London, UK; 5 Department of Infectious Disease Epidemiology, Faculty of Epidemiology and Population Health, London School of Hygiene and Tropical Medicine, London, United Kingdom; 6 Centre for Mathematical Modelling of Infectious Diseases, London School of Hygiene and Tropical Medicine, London, UK; 7 Health Economics Programme, Africa Center for Disease Control and Prevention, Addis Ababa, Ethiopia; 8 Department of Management, Faculty of Business Administration and Management, University of Nigeria Nsukka, Enugu Campus, Nsukka, Nigeria

**Keywords:** Health technology assessment, COVID-19 vaccination, Stakeholders, Decision-making, Nigeria

## Abstract

**Background:**

Nigeria commenced rollout of vaccination for coronavirus disease 2019 (COVID-19) in March 2021 as part of the national public health response to the pandemic. Findings from appropriately contextualized cost–effectiveness analyses (CEA) as part of a wider process involving health technology assessment (HTA) approaches have been important in informing decision-making in this area. In this paper we outline the processes that were followed to identify COVID-19 vaccine stakeholders involved in the selection, approval, funding, procurement and rollout of vaccines in Nigeria, and describe the process routes we identified to support uptake of HTA-related information for evidence-informed policy in Nigeria.

**Methods:**

Our approach to engaging with policy-makers and other stakeholders as part of an HTA of COVID vaccination in Nigeria consisted of three steps, namely: (i) informal discussions with key stakeholders; (ii) stakeholder mapping, analysis and engagement; and (iii) communication and dissemination strategies for the HTA-relevant evidence produced. The analysis of the stakeholder mapping uses the power/interest grid framework.

**Results:**

The informal discussion with key stakeholders generated six initial policy questions. Further discussions with policy-makers yielded three suitable policy questions for analysis: which COVID-19 vaccines should be bought; what is the optimal mode of delivery of these vaccines; and what are the cost and cost–effectiveness of vaccinating people highlighted in Nigeria’s phase 2 vaccine rollout prioritized by the government, especially the inclusion of those aged between 18 and 49 years. The stakeholder mapping exercise highlighted the range of organizations and groups within Nigeria that could use the information from this HTA to guide decision-making. These stakeholders included both public/government, private and international organizations The dissemination plan developed included disseminating the full HTA results to key stakeholders; production of policy briefs; and presentation at different national and international conferences and peer-reviewed publications.

**Conclusions:**

HTA processes that involve stakeholder engagement will help ensure important policy questions are taken into account when designing any HTA including any underpinning evidence generation. Further guidance about stakeholder engagement throughout HTA is required, especially for those with low interest in vaccine procurement and use.

## Background

The first confirmed case of coronavirus disease 2019 (COVID-19) in Nigeria was recorded on 27 February 2020 [1]. During the first wave of COVID-19, the epicentres of infection were in Lagos, Kano and Abuja [2]. A sero-surveillance study conducted in October 2020 in three Nigerian states with 8000 individuals found the prevalence of severe acute respiratory syndrome (SARS-CoV-2) antibodies was 23% in Lagos and Enugu States, 19% in Nasarawa State and 9% in Gombe State [3]. The diversity of SARS-CoV-2 strains indicates multiple introductions of the virus into Nigeria from different parts of the world and adds to evidence of community transmission in different states of Nigeria [4]. As of 7 June 2023, 266,675 confirmed COVID-19 cases and 3155 related deaths have been reported to WHO [5].

Nigeria commenced rollout of vaccination for COVID-19 in March 2021 as part of the national public health response to fight the pandemic [6]. It was expected that mass vaccination would reduce the risk of infection and transmission. At the commencement of the vaccination programme, Nigeria set the ambitious target of vaccinating 40% of the population by December 2021 and 70% by December 2022. This was to be achieved through a four-phase rollout schedule starting with health/frontline workers and strategic leaders (Table 1).

**Table 1:** Summary table of the four phases of vaccine rollout in Nigeria

Phase of vaccine rollout	Targeted population
Phase 1	Health workers, frontline workers, COVID-19 rapid response team, laboratory network, leaders in strategic positions, policemen, petrol station workers and strategic leaders
Phase 2	Older adults ages 50 years and up and those with comorbidities between ages 18 and 49 years
Phase 3	Other people in states and local government areas with high disease burden and those who missed phases 1 and 2
Phase 4	Other eligible populations as vaccines become more available

Financing the cost of COVID-19 vaccination has proven difficult in low- and lower-middle-income countries, including Nigeria. In addition, a sudden increase in government spending coupled with a steep decline in fiscal revenue has caused an economic downturn and financing imbalances in Nigeria [7, 8]. For the first 18 months of the pandemic, there was no significant discussion about the national purchase of COVID-19 vaccines; vaccines used in Nigeria were donated, with national authorities bearing the cost of logistics, training and distribution. However, the Johnson & Johnson COVID vaccine was purchased by the Government of Nigeria on the 11 August 2021 at $7.50 per dose, which is lower than its price of $10 per dose, through African Union’s African Vaccine Acquisition Trust (AVAT) [9]. Plans are in place to continue vaccinating Nigerian citizens and approval has been obtained from The World Bank Board of Directors for a $400 million credit to provide upfront financing for safe and effective COVID-19 vaccine acquisition and deployment within the country [7]; further discussions on vaccine purchases (and associated prices) will be necessary. Findings from evidence-informed health technology assessment (HTA)-type approaches will be important to inform decision-making in this area.

HTA is a framework for collating evidence on healthcare interventions as well as a decision-making framework to inform resource allocation decisions and increase the value of discretionary healthcare expenditures [10, 11]. Cost–effectiveness evidence is key in HTA. Obtaining good value for money for COVID-19 vaccine procurement matters in Nigeria; in a highly resource-constrained setting, opportunity costs are high.

To ensure that evidence generated by the HTA process is taken up into policy, there is a need to involve key stakeholders in the conceptualization of the research (including model development) and the development of recommendations, as well as in communication and policy translation. Without stakeholder engagement, lack of awareness, understanding or confidence about economic models will hinder their use and impact [12]. Studies have also shown that adequate engagement of key stakeholders in evidence generation can lead to increased research uptake [13–16]. Stakeholder involvement can build political and social legitimacy for the evidence-to-policy process, which is particularly important in Nigeria, where the use of research findings by policy-makers and communities has traditionally been limited and challenging [17]. Inadequate communication between researchers and policy-makers, and a lack of involvement of policy-makers and the wider community in shaping research activities, has been observed [18].

In this paper we outline the processes followed to identify COVID-19 vaccine stakeholders involved in the selection, approval, funding, procurement and rollout of vaccines in Nigeria, and describe the process routes identified to support uptake of HTA-related information for evidence-informed policy in Nigeria. This work was part of a larger study that sought to explore the cost–effectiveness of COVID-19 vaccination in Nigeria, taking into account context-relevant policy questions [19]. The Nigerian government had already begun its phase 1 rollout of the COVID-19 vaccine when this work began (phase 1 focused on front-line health workers, etc.); our focus was on phase 2. The economic evaluation analysis we conducted broadly confirmed the “age group prioritization strategy of the Nigerian government (which focused on a 50+ cohort during phase 2 of the rollout) and different types of delivery made little difference to the results” [19].

The work was informed by relevant policy questions from Nigerian decision-makers. Indeed the Nigerian team assembled for this HTA included the involvement of relevant policy-makers in the project from its inception. For instance, represented in this project are members of the Ministerial Expert Advisory Committee on COVID-19 Health Sector Response and the Nigerian Academy of Science, as well as a health economist who serves as the personal assistant to the minister for health, and a member of the Nigerian Immunisation Technical Advisory Group (NGI-TAG). In addition, input into this work was also sought from the African Centre for Disease Control (CDC), and research outputs informed the research underpinning continental-level analyses and guidance. We aim to report on our experience of engaging with COVID-19 vaccine stakeholders in Nigeria. The work is expected to contribute to literature on linkages between policy-makers and research (especially HTA evidence), with COVID-19 specifically in mind, but more generally applicable to other disease areas. This work also provides insights that may be useful in other countries seeking to maximize stakeholder engagement and support evidence-informed policy.

### Methodology

Our approach to engaging with policy-makers and other stakeholders as part of a cost–effectiveness analysis within the HTA process of COVID-19 vaccination in Nigeria was informed by experiences documented previously in Nigeria [16] and consisted of three steps: (i) informal discussions with key stakeholders; (ii) stakeholder mapping, analysis and engagement; and (iii) communication and dissemination strategies for the HTA-relevant evidence produced. We define a stakeholder as individuals, groups or organizations which have a direct interest in the topic under scrutiny and can potentially affect the goals or the performance of a sector, plan or policy [20].

#### Informal discussions with key stakeholders

**Fig. 1:**
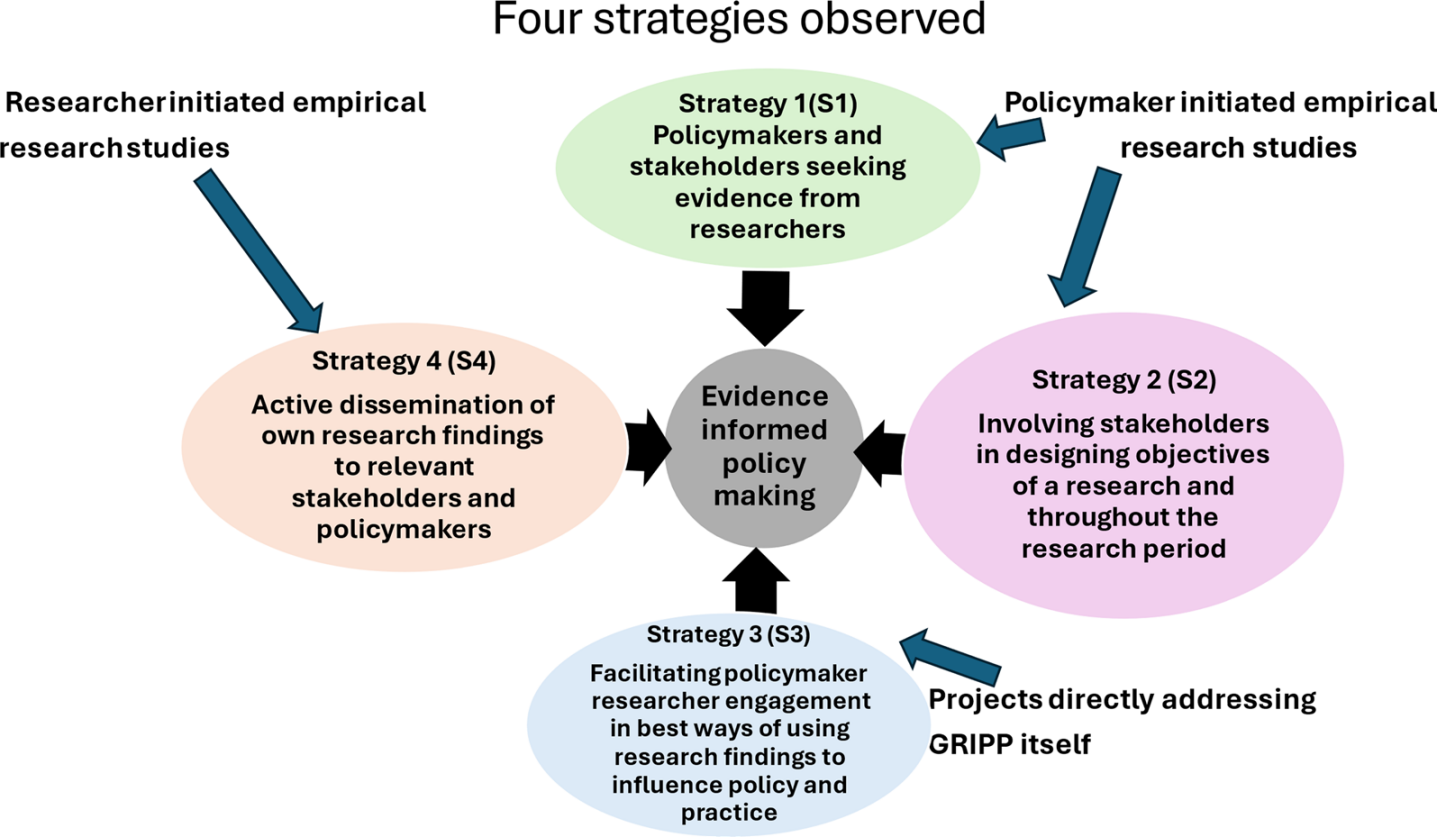
Four evidence-informed policy-making strategies [21]. GRIPP, getting research into policy and practice

The Federal Ministry of Health approached a team of researchers from the Health Policy Research Group of the College of Medicine, University of Nigeria Nsukka (and co-authors on this study), in December 2020, at the peak of the second wave of the COVID-19 pandemic, about the need to produce evidence on the relative cost–effectiveness of different vaccines for the control of COVID-19 in Nigeria. Several informal discussions were held with the Minister of Health’s office, the Nigerian Center for Disease Control (NCDC) and the National Primary Health Care Development Agency (NPHCDA) on the need for such evidence. These stakeholders stressed that such information could support them on investment decisions with respect to COVID-19 vaccines and the wider healthcare system as part of the ongoing response to the pandemic. This method to support engagement with stakeholders in Nigeria on evidence-informed policy-making was based on strategies (1 and 2) described and summarized in Fig. 1 [16].

An informal consultative mechanism was used to reach policy developers and decision-makers (including policy-makers from the Minister of Health’s office, NCDC and NPHCDA), and key stakeholders related to COVID-19 vaccine selection, approval, funding, procurement and rollout in Nigeria. A decision problem formulation process was started, and the opinion of these stakeholders was sought as to what policy questions would guide their vaccine decision-making in the country. This took place over a 3-week period, with key policy questions fine-tuned by the research team to ensure they could be addressed with the available analytical tools and within the timeframe of this study. Further input was then sought from policy-makers and also from stakeholders within the Africa Centre for Disease Control and Prevention (Africa CDC), leading to further adjustments to the selected questions.

#### Stakeholder mapping, analysis and engagement

##### Stakeholder mapping

The researchers identified an initial set of influential stakeholders on the basis of their in-depth knowledge of COVID-19 vaccine selection, approval, funding, procurement and rollout in Nigeria, including those who have formal bureaucratic and political authority to make relevant decisions on COVID-19 vaccination and those who have interest in the outcome of the decision. These stakeholders (*n* = 12 individuals) were comprised of a representative member from each of the following groups: the Presidential Steering Committee (PSC), Federal Ministry of Health (FMoH), NPHCDA, State Primary Health Care Agency/Board, NCDC, Nigeria Immunization Technical Advisory Group (NGI-TAG), legislators, Nigeria Medical Association (NMA), Development Partners, National Agency for Food and Drug Administration and Control (NAFDAC), Ministerial Expert Advisory Committee on COVID-19 Health Sector Response (MEACoC-HSR) and National COVID-19 Vaccine Introduction Technical Working Group.

The identification of a broad variety of pertinent stakeholders was made possible using a snowballing strategy. Initial informants were asked to name people and organizations that had a say in the COVID-19 vaccine selection, approval, funding, procurement and distribution. This technique made it possible to identify both the individuals frequently seen as significant, and those who may have been less well-known or influential but who may nevertheless have an impact on the development and use of vaccines. The strategy used was adapted from elsewhere [21].

The study team then narrowed down the stakeholders on the basis of relevance and role. This took place over 2 weeks. To support further identification of priority stakeholders to help maximize research impact, a categorization scheme was applied on the basis of a qualitative assessment of relative power and interest in the policy area [22]. Here, power is defined as the potential capacity to influence policy decisions, and the power judgment is based on an assessment of stakeholders’ resources [23]. Interest is defined as the degree to which stakeholders are likely to be affected by policy change. The degree of interest or concern stakeholders have about a policy will influence how the stakeholder’s resources, and how much of those resources, will be used in the policy debate [23]. The stakeholders were grouped into four categories depending on their interest and power in COVID-19 vaccine selection, approval, funding, procurement and rollout in Nigeria: (i) high power and high interest; (ii) high power and low interest; (iii) low power and high interest, and (iv) low power and low interest. This provided information on their relative influence and likely interest in the findings of the COVID vaccination cost–effectiveness analysis (CEA), allowing the authors to prioritize stakeholders who would need to be kept informed. In addition, the roles of the stakeholders were identified. The mapping was supported with document reviews.

##### Stakeholder analysis

Stakeholder analysis is the systematic identification, evaluation and prioritization of individuals or organizations who can influence or have an interest in a project or program. [24]. The approach can assist with the development of an effective stakeholder communication and engagement strategy and is a fundamental element of an organization’s stakeholder management plan. We used three key steps as set out elsewhere [23] to conduct the stakeholder analysis, namely:

1. Identifying the groups and individuals (the stakeholders) relevant to the policy issue of focus (in this case cost–effectiveness of COVID-19 vaccines);
2. Determining the current position (in terms of support or opposition) of each stakeholder on the issue;
3. Determining the relative power of each stakeholder over the issue.

To define the power/influence, in the stakeholder analysis, we relied on researchers’ knowledge of the stakeholder’s organizations and through informal discussions with other stakeholders.

##### Stakeholder engagement: crafting the policy questions

Input from Nigerian policy-makers (some of whom are listed in Table 2) was sought through informal emails and discussions, on the key policy questions that need to be addressed to support decision-making in Nigeria and in the Africa CDC at large, with regards to COVID-19 vaccine selection, approval, funding, procurement and rollout activities. These questions were shared with colleagues from the Health Economics Programme at the Africa CDC and the economics and modelling team from the London School of Hygiene & Tropical Medicine (LSHTM). This input was important in helping determine whether the policy questions could be addressed with the modelling tools available.

**Table 2:** Names and roles of the various stakeholders that were mapped

Names	Roles
Presidential Steering Committee on COVID-19 (PSC)	Responsible for reviewing the country’s COVID-19 response. They develop and enforce some policies such as those of restriction. The highest decision-making body on COVID-19 issues including vaccine procurement
Federal Ministry of Health (FMoH)	Takes policy decisions and advises the government on COVID-19 vaccine matters
National and State Primary Healthcare Development Agency (N/SPHCDA)	This organization is responsible for the overall coordination and implementation of the COVID-19 vaccine; for data management, surveillance and microplanning; for vaccine safety, cold chain and logistics; for risk communication; and demand generation
Nigerian Centre for Disease Control (NCDC)	The NCDC makes policies and guidelines with regards to COVID-19 in the country
Nigeria Immunization Technical Advisory Group (NGI-TAG)	An independent expert technical advisory committee that provides guidance to the Ministry of Health and the NPHCDA in making evidence-based immunization-related policy and programme decisions. It is chaired by the permanent secretary of the Ministry of Health
Interagency Coordinating Committee (ICC)	The ICC are the coordinating body for immunization governance in Nigeria
National Agency for Food and Drug Administration and Control (NAFDAC)	The NAFDAC are responsible for approving vaccines for use in Nigeria, and they also monitor its safety. They issue pre-arrival regulatory approvals (import permit), and on arrival, they take samples from each batch for analysis to ensure the quality is of global standards
National COVID-19 Vaccine Introduction Technical Working Group	A multi-sectoral COVID-19 Vaccines Introduction Technical Working Group (TWG) to ensure readiness for timely COVID-19 vaccine introduction in the country. Members of the TWG include stakeholders involved in COVID-19 response. They develop the National Vaccine Deployment Plan (NVDP) and monitor preparedness for rollout of the COVID vaccine
National legislators	Responsible for giving legal backings to policies
Ministerial Expert Advisory Committee on COVID-19 Health Sector Response (MEACoC-HSR)	This is a high-level advisory platform established by the Minister of Health to provide technical advisory support to the leadership of the health sector’s COVID-19 response in Nigeria
Coalition Against COVID-19 (CACOVID)	A private sector task force in partnership with the federal government, the Nigeria Centre for Disease Control (NCDC) and the WHO with the sole aim of combating coronavirus (COVID-19) in Nigeria. They are tasked with pulling resources across industries to provide technical and operational support while providing funding and building advocacy through aggressive awareness drives. They are partnering with NPHCDA to activate a comprehensive logistic COVID-19 vaccine deployment plan at a sub-national level
National Council on Health	The highest decision-making body for health issues in Nigeria
Nigerian Academy of Science	They assess Nigeria’s response to COVID-19 and give expert opinion which is highly respected Pointing out the areas of learning and the areas where more is needed
Professional bodies	They advise the government on COVID-19-related issues
Federal Ministry of Finance, Budget and National Planning	They work with the Ministry of Health and the budget office of the federation to cost the required vaccine and approve and release funds for its purchase
Development partners	Provide funds and technical advice for the purchase of COVID-19 vaccines
Regional bodies	Provide advice to the government of Nigeria on COVID-19 vaccine purchase
Research forums	They carry out research related to COVID-19 vaccines and make local information available for policy-makers’ consumption to guide evidence-based decision-making
The media	Translation of complex messages and an important enabler for the advocacy process by holding public events and disseminating research evidence

#### Communication and dissemination to support uptake of the HTA evidence

A team of Nigerian experts was established to collect evidence on relevant economic evaluations and HTA studies on vaccines and assess their actual use in decision-making processes. There was involvement of relevant policy-makers in the project from its inception. For instance, members of the MEACoC-HSR, the Nigerian Academy of Science, a health economist from the office of the Minister of Health and a member of the Nigerian, NGI-TAG, were key members of the project. This team continued discussions with the Minister of Health’s office, NCDC and NPHCDA on the study progress. As research partners in this study, a research link was established between the LSHTM-Nigeria research team and the Africa CDC; the latter also provided input into the policy and research questions identified for this study.

## Results

### Stakeholders mapping, analysis and engagement

The stakeholder mapping exercise highlighted the range of organizations and groups within Nigeria that could use the information from the HTA to guide decision-making. It was anticipated that these organizations would benefit from the findings of the study, either in costing their programs or in the use of the evidence for policy and decision-making and as a tool for advocacy. The results may also offer useful data and opportunities for resource allocation, strengthening of the health system and knowledge translation, resulting in improved health outcomes and better-informed decision-making. The names, roles and responsibilities of the various stakeholders that were mapped are shown in Table 2.

**Fig. 2:**
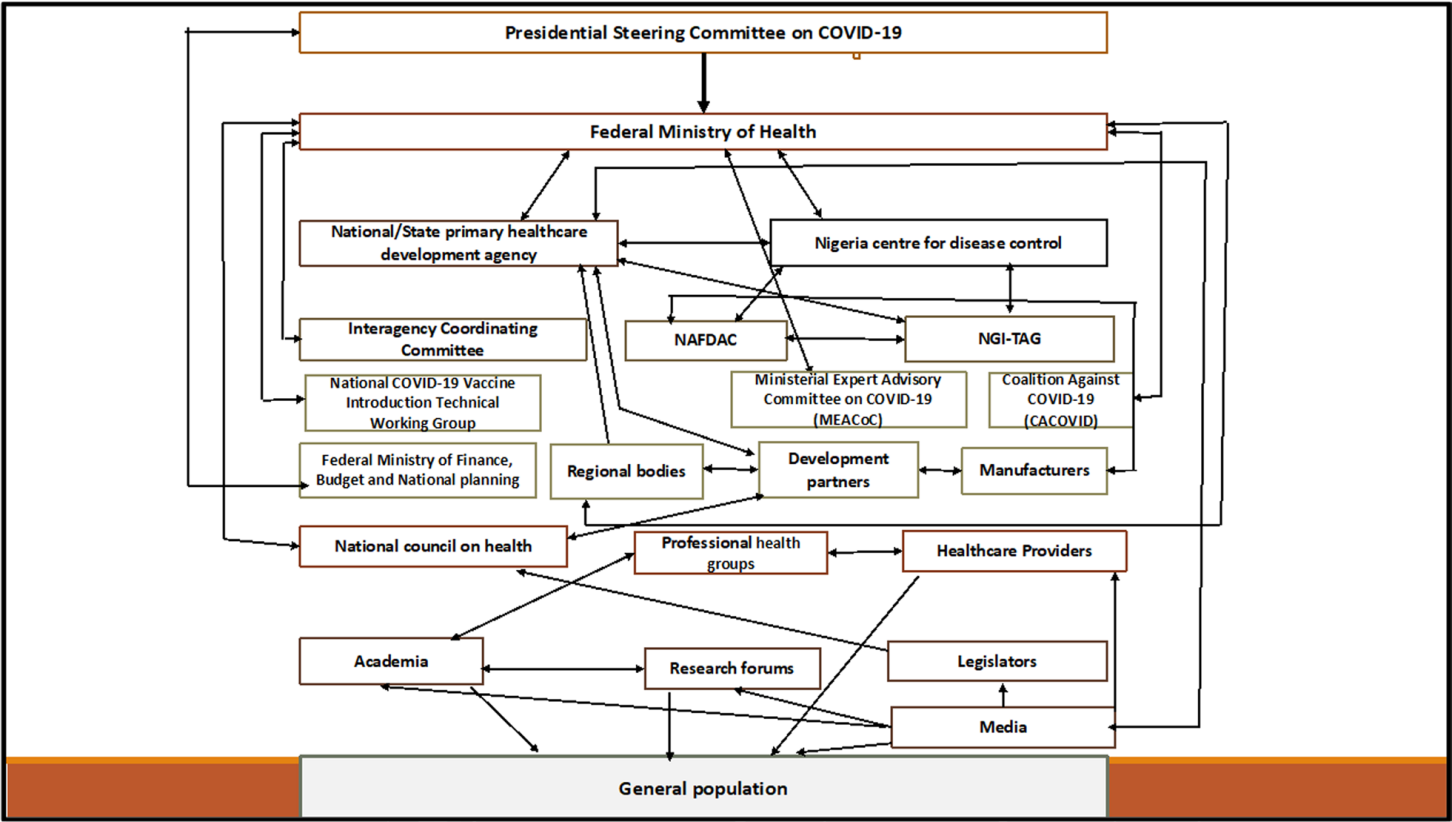
Interaction of actors involved in decision-making on vaccine procurement

Figure 2 illustrates key stakeholders involved with the decision-making of COVID-19 vaccine procurement and their interaction with one another. They either make the decisions or are impacted by the decisions made. For example, the Presidential Steering Committee (PSC) reports directly to the President of Nigeria. The PSC interacts with the FMoH, which is responsible for developing vaccine policy for the country and ensuring implementation. The FMoH interacts with all the other agencies, as shown in Fig. 2. The NPHCDA receives and coordinates the distribution of the vaccines until it gets to the end users and, with the FMoH, jointly decides on the types of vaccine to be procured and the quantity needed, as well as on issues linked to storage capacity. The NAFDAC regulates and licences potential vaccines prior to use. Figure 2 also shows other important actors, including the various technical working groups, development partners (DPs) and regional bodies.

It is important to highlight that the NGI-TAG provides evidence-based recommendations to the NAFDAC, NCDC and NPHCDA. The Federal Ministry of Finance (FMoF) provides funds for the procurement, and the regional bodies and DPs liaise with the manufacturers for bulk purchase at reduced prices. The National Council on Health (NCH), professional health groups and healthcare providers provide feedback via various organizations to the FMoH. This feedback helps determine the type of vaccines to procure. Finally, stakeholders, such as academia, research forums and the media, support the production and dissemination of evidence for use by policy-makers, including adverse events from vaccines. The general population represent the end users, that is, the groups that will receive the procured vaccines.

**Fig. 3:**
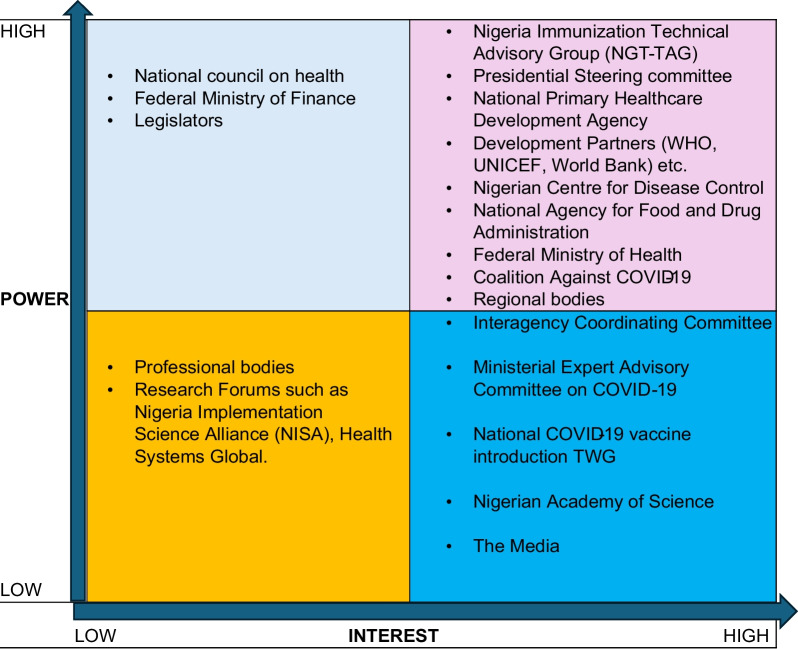
Power/interest grid of COVID-19 vaccine selection, approval, funding, procurement and rollout in Nigeria

The stakeholders in Fig. 2 were categorized using a power/interest grid framework [23], as shown in Fig. 3.

The resulting matrix (Fig. 3) identified key stakeholders who could assist with the prioritization of resources within the context of COVID-19 vaccination and as such provided a foundation for a communication and engagement plan. Thus, the stakeholders were categorized into four groups:

1. High power and high interest (upper right quadrant): These are the most important stakeholders and are mainly the decision-makers, having the biggest impact on the project’s success. They were considered a high priority for obtaining feedback on the HTA results as well as the conduct of the HTA.
2. High power and low interest (upper left quadrant): Because of their influence in Nigeria, we also prioritized these stakeholders given the power that they could wield, and we aimed to keep them abreast with the evidence despite their relatively low interest.
3. Low power and high interest (lower right quadrant): We aimed to keep these stakeholders informed of the evidence to maintain their high interest in the HTA.
4. Low power and low interest (lower left quadrant): This group was considered a low priority for communication and dissemination activities.

### Development of research questions

Initially, a list of policy-relevant questions that could be considered in a CEA/HTA were drafted:

1. Which COVID-19 vaccines should be bought?
2. How much of each vaccine should be bought?
3. What is the maximum price to pay for each specific vaccine?
4. What is the best way to deliver vaccines (for example, fixed posts at health facilities, campaigns or targeted campaigns)?
5. Which enabling interventions can best support vaccination?
6. How much funding can and should be allocated to support uptake and address vaccine hesitancy, and to which groups (for example, young people, ethnic minorities, women, certain religions and those from socially deprived backgrounds)?

Taking into account the broad policy questions described above, the researchers and key stakeholders agreed that the focus for the de novo CEA within the HTA should be framed around three analytically feasible questions:

1. Which COVID-19 vaccines should be bought, and what is the maximum price to pay?
2. What is the best way to deliver the vaccines?
3. What are the cost and cost–effectiveness of vaccinating people aged 18–49 years?

These three policy-relevant questions were further expanded by the researchers through an interactive and consultative process into the context-specific decision problem, as set out in Table 3. This was used as the basis for modelling the cost–effectiveness of the selected vaccines.

**Table 3:** Summary of the decision problem(s) modelled in the University of Nigeria Nsukka-LSHTM analysis

Intervention	COVID-19 vaccination, specifically the use of the following vaccines: viral vector vaccines, similar to Oxford/Astra-Zeneca (AZ) and Johnson & Johnson (JJ) mRNA vaccines, similar to Moderna & Pfizer-BioNTech
Comparator	No vaccination scenario
Perspective	Health system
Delivery mechanisms	● Health Facility (HF) ● Campaign (C) ● Targeted Campaign (TC)
Age groups	All adults, 50+ year olds, 18–49 year olds
Coverage	25–100%

Table 3 shows the summary of the decision problems modelled in the study. The epidemiological model of virus transmission parametrized with Nigeria-specific data shown in Table 3 was combined with a costing model that incorporated local resource use assumptions and prices, both for vaccine delivery as well as costs associated with care and treatment of COVID-19. Scenarios of vaccination were compared with no vaccination [19].

### Communication and dissemination to support the uptake of the HTA evidence

For the purpose of this HTA, the following outline dissemination plan was developed and used to support dissemination in an ongoing process (Table 4):

- 1. Final results (full HTA report) presented to the Minister of Health and PSC on COVID-19.
- 2. Production and distribution of policy briefs and blogs for stakeholders, particularly decision-makers to enable decisions to be taken on the basis of scientific evidence, and appropriate sustainable actions to be undertaken. We used this type of evidence format because some of the policy-makers might be non-specialized audiences but still need to make decisions about vaccine procurement.
- 3. Presentation at the National Council on Health meetings (the highest decision-making body on health).
- 4. Organizing policy dialogues with decision-makers in the FMoH, FMoF, NCDC, NPHCDA, NHIS, NGI-TAG and PSC. Policy dialogues will raise the profile of vaccine procurement and promote relationships between these stakeholders.
- 5. Co-production: preparation of peer-reviewed articles and blogs (as necessary) in collaboration with members of the team and policy-makers.
- 6. Presentation at the conference of the Association of Public Health Physicians of Nigeria at the annual scientific meeting of the West African College of Physicians, and other local conferences that will come up in the country.

**Table 4:** Dissemination of the research output: meetings, events and publications

Month/year	Name/location	Influential attendees (name, position and organization)	People in attendance (total)	What messages were shared
October 2021	A virtual webinar on the role of modelling and economic evidence to inform COVID-19 vaccine procurement and distribution strategies	John N. Nkengasong director of the Africa CDC and Prevention Donald Kaberuka, AU special envoy on COVID-19 and AU high representative for Peace Fund Financing the Union and Health Financing Javier Guzman, director of Global Health Policy and a senior fellow at the Center for Global Development John Ataguba, director of the Health Economics Unit at the University of Cape Town and deputy director of Africa Health Economics and Policy Association (AfHEA) Ahmed E. Ogwell, deputy director of Africa CDC Edith Kouassi, technical advisor to the Minister of Health, Public Hygiene and Universal Health Coverage, Côte d’Ivoire Lucy Mecca, head of the National Vaccines and Immunization Program, Kenya Ministry of Health	25	Nigeria COVID-19 vaccine HTA preliminary results to stakeholders; power point presentation
December 2021	University Of Nigeria, Nsukka	Researchers in health policy and systems comprising health economists, public health physicians, sociologists and health administrators	30	Nigeria COVID-19 Vaccine HTA preliminary results to stakeholders
February 2022	Nigeria	Representatives from the Federal Ministry of Health, National Immunization Technical Advisory Group and the Nigeria CDC	30 policy-makers	Nigeria’s COVID-19 final report and policy brief disseminated
March 2022	Nigeria	General public	General public	Policy Brief: Benjamin S.C. Uzochukwu, Chinyere Okeke, Sergio Torres-Rueda, Carl Pearson, Eleanor Bergren, Anthony McDonnell, Anna Vassall, Mark Jit and Francis Ruiz. Understanding the cost–effectiveness of COVID-19 vaccination in Nigeria. A policy brief, 2022 https://www.cgdev.org/sites/default/files/understanding-cost-effectiveness-covid-19-vaccination-nigeria.pdf
April 2022	Nigeria, Ethiopia, Kenya and Africa CDC	General public	General public	Blog: Lydia Regan, Stacey Orangi, Edwine Barasa, Benjamin Uzochukwu, Chinhere Okeke, Firmaye Bogale, Elias, Asfaw, Anna Vassall, Francis Ruiz, Anthony McDonnell, Pater Baker, Javier Guzman, Justice Nonvignon and Tom Drake. Price, priorities, pace: Three factors that drive cost–effectiveness of COVID-19 vaccination strategies in Kenya, Nigeria and Ethiopia. Blog Post, April 2022. https://www.cgdev.org/blog/price-priorities-pace-three-factors-drive-cost-effectiveness-covid-19-vaccination-strategies
March 2022	Nigeria. 38th Annual General Meeting and Scientific Conference of the Association of Public Health Physicians of Nigeria. Ilorin,	Public health physicians in Nigeria and some invitees	Over 250 registered	Health technology assessment of COVID-19 vaccines in Nigeria, results
April 2022	Enugu State Primary Healthcare Development Agency	The executive secretary and his team		Policy brief and report
April 2022	Nigeria; 2022 NCRC Research Colloquium	A wide range of stakeholders in the health sector, researchers across more than 20 institutions from governmental and non-governmental organizations within and outside Nigeria, partners, policy-makers, NCDC and media	200	Health technology assessment of COVID-19 vaccines in Nigeria, results
July 2022	45th Annual General and Scientific Conference of the West African College of Physicians, Nigerian Chapter, Enugu	Fellows and members from all over the country and outside, policy-makers and partners	450 registered and attended either physically and virtually	Health technology assessment of COVID-19 vaccines in Nigeria, results
26 October 2022	National Primary Health Care Development Agency	The Executive Director/CEO of NPHCDA		The policy brief and report
March 2023	LSHTM and University of Nigeria	General public	General public	Peer-reviewed publication Ruiz FJ, Torres-Rueda S, Pearson CAB, Bergren E, Okeke C, Procter SR, Madriz-Montero A, Jit M. Vassall and Uzochukwu BSC. (2023) What, how and who: Cost–effectiveness analyses of COVID-19 vaccination to inform key policies in Nigeria. PLOS Glob Public Health 3(3): e0001693 https://doi.org/10.1371/journal.pgph.0001693

Table 4 shows the dissemination of the results with the different messages that were shared with different stakeholders on the basis of the stakeholder analysis done. The stakeholders included academia, policy-makers from both the national and sub-national levels, and the Africa CDC. The results were well received by these stakeholders, and it is still ongoing.

## Discussion

In this study, we report on our experience of engaging with COVID-19 vaccine stakeholders in Nigeria involved in the selection, approval, funding, procurement and rollout of vaccines and describe the process routes to support policy uptake of HTA-related findings. Our study contributes to the literature linking policy-makers to research (especially cost–effectiveness evidence) with COVID-19 specifically in mind but is also applicable to other disease areas. This work could help inform the development of an HTA mechanism in Nigeria, which is currently absent. In addition, it also provides learning for other countries on how to maximize stakeholder engagement and garner support for evidence-informed policy.

The importance of decision-makers comprehending HTA requirements, uses and scope as well as the optimal approaches to incorporating HTA components into the design of decision-making mechanisms has been emphasized by a number of authors. [25]. HTA is not a narrow technocratic exercise, and as such it is important to also identify or develop methods of wider citizen and stakeholder involvement that could be considered best practice internationally. For example, the evidence-informed deliberative processes (EDPs) framework [26]), though not used in our research due to limited resources and time, can support the decision-making process in HTA bodies and, thus, contribute to the legitimacy of recommendations and decisions. How any EDP process is implemented as part of HTA institutionalization in a particular setting would likely be shaped by local conditions and preferences.

Although stakeholder involvement is key to improving the HTA process, the form and timing of such improvements must be adapted to local contexts and data. In Nigeria, the perceived availability and accessibility of suitable local data to support HTA varies widely but is mostly considered inadequate and limited [27]. In our study, it was necessary to ensure that the cost–effectiveness analyses were consistent with the epidemiological context and that stakeholders were involved in (at least) initial discussions around study design to enhance credibility and engagement [28]. Inadequate understanding of the links between HTA-related outputs and decision-making can lead to key stakeholder exclusion and loss of policy relevance [29]. To maximize the relevance and timeliness of COVID-19 vaccine HTAs (or indeed HTAs for any topic), it is important to first identify the stakeholders and processes for decision-making on technology procurement [30].

Given the large number of stakeholders in COVID-19 vaccine procurement and use, it was necessary to identify priority actors to guide the dissemination of research findings. As noted by some authors, this process should not be considered as a one-off activity, but rather an ongoing pattern of engagement, to allow for changes in the stakeholders’ positioning [24]. Overall, the power/interest matrix is a useful tool for stakeholder analysis since it offers a structured method for comprehending stakeholder dynamics, setting priorities for engagement initiatives, and successfully managing relationships. However, other criteria could also be considered when grouping stakeholders, such as influence, legitimacy, expertise, attitude and resources, which could provide a more nuanced understanding of stakeholders and help to inform engagement strategies that are tailored to their needs and concerns [31].

The experience set out in the present study demonstrates the importance of identifying key stakeholders early on as part of any evidence-driven activity and ensuring an ongoing degree of engagement to enhance the value of the research findings. Despite the depth of the stakeholder mapping, issues with power dynamics and representation of stakeholder interests are regularly encountered when trying to acquire a true reflection of the balance of all stakeholders. [32, 33]. Ensuring representation of stakeholder interests, power balance and transparency of decision-making are critical in COVID-19 vaccine procurement and use in Nigeria. However, it is difficult to say how this played out in our study, as there are multiple stakeholders involved, and the situation is constantly evolving. We found it easier to engage some stakeholder groups such as government workers and academia, than those in the private sector such as the CACOVID in this study. Even though efforts have been made to involve stakeholders and ensure transparency, there is always room for improvement, such as inclusive stakeholder representation, early and ongoing engagement, establishing mechanisms for two-way communication, allowing stakeholders to provide feedback and making decisions that are informed by the input of all stakeholders. Ongoing communication and engagement with stakeholders are crucial to addressing their concerns and ensuring accountability. Researchers need concrete information on potential stakeholder groups affected by COVID-19 immunization to be more inclusive. Nevertheless, structured guidance on how to maximize stakeholder involvement in HTA is needed.

Manufacturers of vaccines were not included as a stakeholder group in this work for two reasons. First, we focused on an independent assessment of the cost–effectiveness of COVID-19 vaccination that could be used by the FMoH and others in a wider procurement process that could involve discussions with the industry. Secondly, we deliberately did not model specific products, describing our interventions instead as viral vectors or mRNA vaccines. Modelling of specific products would have certainly lengthened the assessment of vaccine effectiveness and introduced industry consultation steps that would have required active approval and leadership from relevant Nigerian authorities.

Identifying a long list of policy-relevant questions informed by discussion with Nigerian decision-makers was seen as necessary to enhance uptake and support meeting expectations set out in early discussions. In addition, our findings are also anticipated to assist Africa CDC in their planning around the role of cost–effectiveness evidence in vaccine rollouts generally, and the development of HTA-informed decision-making processes to inform resource allocation decisions.

Policy-makers in this study highlighted the importance of supply chain consequences, especially with cold chain, and storage when decisions are made on vaccine selection. It is well known that investing in vaccinations to prevent COVID-19 infections might have a significant return on investment. [34]. However, there could be very significant budgetary or resource consequences for the government and other payers from very rapid vaccine rollouts, especially where there are important cold chain requirements. Formal cost–effectiveness analysis combined with an assessment of budget impact features typical of the HTA process are clearly needed to support appropriate vaccine selection in a given context. Information regarding the highest price charged for each COVID vaccine choice was also requested by policy-makers.

Policy-makers were interested in knowing what vaccines, and critically at what price, should be purchased. However, in addition, policy-makers were concerned about the costs of delivery to inform the best way to deliver each of the vaccines. The aim here was to support policy-makers in deciding which method provides more cost–effective coverage, considering the high level of hesitancy observed in the uptake of the COVID vaccine [35–37].

The policy question regarding the cost and cost–effectiveness of immunizing those aged 18 to 49 years with or without underlying diseases was significant in guiding government decisions on whether to implement this prioritization approach. It is especially important to be clear about the decision problem and the evidence-to-policy process from the outset in urgent or high-stakes situations such as pandemics to ensure that a range of information is taken into account and avoid misunderstandings or delays.

It is pertinent to note that, prior to the delivery of the cost–effectiveness study carried out in response to policy-maker requests, the primary choices about vaccine deployment had been made in Nigeria. However, the results were crucial in reaffirming the key choices made regarding age prioritization and delivery strategies and in giving a framework for how comparable issues can be dealt with in the future.

As shown in our study, a strategy for communication and dissemination should be informed by a stakeholder mapping exercise. Arguably the more important a stakeholder is (in terms of power and interest for example), the more important it is to establish an effective means of communication with that stakeholder. Timeliness is also an important consideration, and it is a challenge for HTA systems everywhere. Findings need to be delivered quickly (especially in pandemic contexts) and in a style that is clear and useful to the target group. Consequently, policy and decision-makers must be included in the study process. Additionally, it is important to share plain-language research summaries through a variety of platforms, such as social media and in-person meetings between researchers and end users [38, 39].

Aside from the production of a detailed full HTA report on COVID-19 vaccination in Nigeria, other dissemination materials were produced and disseminated by the study team, including a policy brief [40], blogs [41, 42], conference presentations and a peer-reviewed publication [19]. These products were tailored to the needs of the various stakeholders and delivered at different time points in the study.

We found that modelling results can only be effective in shaping policy when they are pertinent to the most important policy-based decision problems and are effectively communicated. However, to prepare the results for dissemination to policy-makers, some authors have suggested essential items that modellers should disclose before making any policy suggestions such as models’ objectives, methods, assumptions, data sources and fitness-for-purpose in context, and whether they actively involved decision-makers and policy-makers to determine their needs.

The results of this study could have significant implications for the development of policies and the improvement of public health in Nigeria, especially in the context of managing future pandemics. The study’s primary objective of determining the optimal COVID-19 vaccines for procurement holds significant importance for the Nigerian government. To optimize resource allocation and ensure the population receives the most effective protection against the virus, it is important to understand the most relevant vaccinations based on variables such as efficacy, safety and cost–effectiveness, given the wide range of alternatives available. This has been noted by Santoli et al., who noted that economic evaluation data for mass vaccination is crucial for decision-makers to make evidence-based, value-based decisions to ensure equitable access and reduce the global COVID-19 burden [43].

The stakeholder mapping exercise done during the study emphasizes the significance of engaging a diverse array of organizations and people in decision-making processes. Incorporating the participation of public/government, private and international organizations guarantees the inclusion of a wide range of viewpoints, hence enhancing the probability of formulating policies and plans that are pertinent, agreeable and efficient across different sectors of society. This has been echoed in a study that addresses the under-researched issue of stakeholder identification and engagement in problem-structuring interventions [44].

The devised dissemination plan for the study, encompassing the disclosure of comprehensive HTA outcomes, the creation of concise policy briefs and the delivery of presentations at both national and international conferences, is crucial for effectively converting research findings into implementable policies and practices. The study’s wide dissemination of findings can have a significant impact on decision-making processes at several levels and facilitate evidence-based policy formulation not only in Nigeria but also in other regions. The study highlights the significance of involving stakeholders in HTA procedures to guarantee the examination of pertinent policy inquiries and the production of supporting evidence. Offering advice on how to engage stakeholders effectively, especially those who are not very interested in vaccine procurement and usage, will improve the inclusivity and efficacy of future HTA programmes. This, in turn, will strengthen health systems and lead to better public health outcomes. Uzochukwu et al. (2020) noted that stakeholder participation in identifying HTA topics and conducting relevant research will enhance the use of HTA evidence produced for decision-making [27].

In summary, the study’s results can be used to shape policy-making and public health practices in Nigeria. This includes making informed decisions about COVID-19 vaccine procurement, delivery and cost–effectiveness. Additionally, the study encourages stakeholder involvement and evidence-based decision-making in future health technology assessments. The importance of the research, especially in managing future pandemics in the country cannot be over-stressed.

As this paper set out to to identify COVID-19 vaccine stakeholders involved in the selection, approval, funding, procurement and rollout of vaccines in Nigeria, and describe the process routes to support uptake of HTA-related information for evidence-informed policy in Nigeria, and given the study’s findings and the policy problems that have been identified, we provide some practical recommendations and consequences for policy and practice:

1. Utilize health technology assessments (HTAs) and cost–effectiveness studies in collaboration with key stakeholders to inform decisions regarding vaccination acquisition and prioritization in the country.
2. Policy-makers should evaluate the economic and social ramifications of vaccination techniques, guaranteeing that policies promote both public health and economic revitalization.
3. Facilitate coordination among public/government, commercial and international organizations to guarantee a synchronized approach to vaccine procurement, delivery and monitoring. It is therefore essential to involve stakeholders throughout the decision-making process to improve openness, establish agreement and resolve issues around vaccine acquisition and utilization and secure their buy-in and enhance getting research into policy practice.
4. Develop and execute a comprehensive strategy to effectively communicate the HTA findings to important stakeholders in a prompt and easily understandable way. This entails generating policy briefings that succinctly outline significant discoveries, delivering presentations of findings at both domestic and global conferences and publishing articles that have undergone peer review to promote the dissemination of knowledge and enable well-informed decision-making.
5. Deliver training and guidance on efficient methods of engaging stakeholders, specifically targeting individuals with limited interest in vaccine procurement and utilization. Empower stakeholders with the necessary expertise and resources to actively engage in HTA processes, promoting a cooperative and all-encompassing approach to making decisions on the basis of evidence.

Finally, to enhance the efficacy of COVID-19 vaccination endeavours in Nigeria, officials might apply these suggestions to optimize resource allocation and fortify the country’s entire response to the pandemic. Additionally, fostering stakeholder engagement and knowledge sharing can build trust, promote accountability and facilitate sustainable health system improvements beyond the current crisis.

### Limitations of the study

The sampling of stakeholders was not a nationally representative sample. The analysis might not have a complete picture of the many perspectives and interests across various backgrounds and sectors in Nigeria. As a result, there may be an incomplete picture of the stakeholders involved in COVID-19 vaccine selection, approval, funding, procurement and rollout in Nigeria and their possible impact on the process, which could lead to resistance or mistrust from stakeholders who believe their opinions are not adequately represented. However, our sample of policy-makers and stakeholders was not intended to be representative of the whole country, but rather based on those with in-depth knowledge of COVID-19 vaccine selection, approval, funding, procurement and rollout in Nigeria. Also, to mitigate this limitation, the study provided a clear rationale for the selection criteria of policy-makers and stakeholders, that is, those who were involved in vaccine selection, etc. Also, the qualitative insights gained from the interviewed stakeholders supported our approach.

The roles of the media and community members in the HTA process were not assessed. It is possible that important information about public opinion and expectations was overlooked by not consulting the media. Without involving the community, important clues about their issues can go unnoticed. Engaging the community and the media encourages cooperation and stakeholder buy-in. In general, the absence of the media and the community from the stakeholder analysis may restrict the scope and depth of the stakeholder understanding, and may hinder the decision-making process and buy-in from important stakeholders. This will form a basis for a further study.

There was no mention and analysis specifically done for those living in difficult terrains, security-compromised areas of Nigeria and those living with physical, intellectual and developmental disabilities. These are important groups that we were unable to explore in the present work because of time and data constraints. Extensive additional analytic work would be needed (including primary data collection) to gather the evidence necessary to parametrize a model to consider the cost–effectiveness of vaccination for these different groups and any support programmes that may be needed (that is, to improve access). This was not considered feasible within the time frame of the HTA. However, authors can take this gap and explore filling it in future studies.

## Conclusions

HTA provides an evidence-informed framework for guiding decisions on the adoption and rollout of health interventions. An important aspect of HTA is ensuring key stakeholder engagement in the selection of policy choices subject to any analysis. Based on the authors’ experience with evaluating COVID-19 vaccination in Nigeria, including the conduct of a locally relevant cost–effectiveness analysis, stakeholders’ experience and knowledge can be used to pinpoint important policy issues, which in turn helps determine the emphasis of any HTA and ensures its usefulness for policy. Engaging stakeholders throughout the process from formulating policy questions and helping shape the study design, to the final dissemination of results, is a necessary part of an evidence to policy framework such as HTA.

HTA serves as a link between those responsible for developing policy and those who can provide the evidence needed to inform those policy choices. It is not a purely technical exercise limited to the delivery of cost–effectiveness analyses. Stakeholder engagement in HTA approaches will guarantee that significant policy concerns are taken into account when identifying and generating the required evidence. To inform decision-making processes and support any future pandemic response, in Nigeria and sub-Saharan Africa, researchers in Nigeria and Africa and policy-makers may need more direction on stakeholder participation approaches when utilizing HTA-based approaches.

## Data Availability

Not applicable.

## Abbreviations

Africa CDC: Africa Centre for Disease Control and Prevention
CACOVID: Coalition against COVID-19
CEA: Cost–effectiveness analysis
FMoH: Federal Ministry of Health
HTA: Health technology assessment
MEACoC-HSR: Ministerial Expert Advisory Committee on COVID-19 Health Sector Response
NAFDAC: National Agency for Food and Drug Administration and Control
NCDC: Nigerian Centre for Disease Control
NGI-TAG: Nigerian Immunisation Technical Advisory Group
NPHCDA: National Primary Health Care Development Agency
NVDP: National Vaccine Deployment Plan
TWG: Technical Working Group
